# Sociodemographic and clinical characteristics of treated and untreated adults with bulimia nervosa or binge-eating disorder recruited for a large-scale research study

**DOI:** 10.1186/s40337-023-00846-4

**Published:** 2023-07-31

**Authors:** Emily A. Carrino, Rachael E. Flatt, Pratiksha S. Pawar, Christina M. Sanzari, Jenna P. Tregarthen, Stuart Argue, Laura M. Thornton, Cynthia M. Bulik, Hunna J. Watson

**Affiliations:** 1 Department of Psychology and Neuroscience, University of North Carolina at Chapel Hill, Chapel Hill, NC USA; 2 Department of Psychiatry, University of North Carolina at Chapel Hill, Chapel Hill, NC USA; 3 Department of Biotechnology, Dr D. Y. Patil Biotechnology and Bioinformatics Institute, Dr. D. Y. Patil Vidyapeeth, Pune, India; 4 Department of Psychology, State University of New York at Albany, Albany, NY USA; 5 Recovery Record, Inc, Palo Alto, CA USA; 6 Department of Nutrition, University of North Carolina at Chapel Hill, Chapel Hill, NC USA; 7 Department of Medical Epidemiology and Biostatistics, Karolinska Institutet, Nobels Väg 12A, 171 65 Solna, Stockholm Sweden; 8 Discipline of Psychology, School of Population Health, Curtin University, Perth, Australia; 9 Division of Paediatrics, School of Medicine, The University of Western Australia, Perth, Australia

**Keywords:** Binge Eating Genetics Initiative (BEGIN), Binge-eating disorder, Bulimia nervosa, Diversity, Health inequality, Level of care, Treatment access, Treatment gap, Treatment-seeking

## Abstract

**Background:**

Eating disorders affect millions of people worldwide, but most never receive treatment. The majority of clinical research on eating disorders has focused on individuals recruited from treatment settings, which may not represent the broader population of people with eating disorders. This study aimed to identify potential differences in the characteristics of individuals with eating disorders based on whether they self-reported accessing treatment or not, in order to contribute to a better understanding of their diverse needs and experiences.

**Methods:**

The study population included 762 community-recruited individuals (85% female, M ± SD age = 30 ± 7 years) with bulimia nervosa or binge-eating disorder (BN/BED) enrolled in the Binge Eating Genetics Initiative (BEGIN) United States study arm. Participants completed self-report surveys on demographics, treatment history, past and current eating disorder symptoms, weight history, and their current mental health and gastrointestinal symptoms. Untreated participants (*n* = 291, 38%) were compared with treated participants (*n* = 471, 62%) who self-reported accessing BN/BED treatment at some point in their lives.

**Results:**

Untreated participants disproportionately self-identified as male and as a racial or ethnic minority compared with treated participants. Treated participants reported a more severe illness history, specifically, an earlier age at onset, more longstanding and frequent eating disorder symptoms over their lifetime, and greater body dissatisfaction and comorbid mental health symptoms (i.e., depression, anxiety, ADHD) at the time of the study. A history of anorexia nervosa was positively associated with treatment engagement. Individuals self-reporting a history of inpatient or residential treatment exhibited the most severe illness history, those with outpatient treatment had a less severe illness history, and untreated individuals had the mildest illness history.

**Conclusions:**

Historically overlooked and marginalized populations self-reported lower treatment access rates, while those who accessed treatment reported more severe eating disorder and comorbid mental health symptoms, which may have motivated them to seek treatment. Clinic-based recruitment samples may not represent individuals with milder symptoms or racial and ethnic diversity, and males. Community-based recruitment is crucial for improving the ability to apply research findings to broader populations and reducing disparities in medical research.

*Trial Registration* ClinicalTrials.gov NCT04162574 (https://clinicaltrials.gov/ct2/show/NCT04162574).

**Supplementary Information:**

The online version contains supplementary material available at 10.1186/s40337-023-00846-4.

## Abstract (plain language summary)

The majority of individuals with eating disorders never enter treatment. However, most clinical research on eating disorders recruits participants from clinics and treatment centers. Therefore, most of our knowledge about eating disorders may not represent the majority of people with eating disorders, particularly those who do not enter treatment. We studied 762 people with bulimia nervosa or binge-eating disorder recruited from the community to a large research study. We compared participants who reported never accessing treatment (38%) to participants who reported having accessed treatment at some point in their lives (62%). Untreated participants were much more likely to identify as male and as a racial or ethnic minority compared with participants who had accessed treatment (who identified mostly as female and White). Participants who had accessed treatment had a more severe illness history and higher levels of body dissatisfaction and mental health symptoms at the time of the study. The present study highlights the importance of recruiting research participants from the community to clinical studies as a way to address medical inequity in marginalized and underrepresented groups. Additionally, caution is advised when generalizing research findings from research samples who have sought treatment to all people with eating disorders.

**Supplementary Information:**

The online version contains supplementary material available at 10.1186/s40337-023-00846-4.

## Background

Few individuals with eating disorders—only 23% (95% CI = 17%, 31%)—access treatment [1]. Published guidelines by professional groups acknowledge the importance of early intervention for eating disorders on health outcomes [2]. The factors associated with non-treatment, and the comparability between those who have and have not sought treatment, are not well understood. Historically, research on eating disorders has typically been conducted on clinic-based samples. Contemporaneously, register-based studies with clinic-based samples identified through electronic medical records are becoming increasingly common and confer many advantages. Some are very large, with case–control sample sizes now in the millions, and such findings could be very influential; however, they only capture individuals detected by the healthcare system [3]. Limited consideration has been given to the implications of sample provenance in clinical eating disorder research. In the present study, we compared the features of research participants with binge-type eating disorders (bulimia nervosa and/or binge-eating disorder; BN/BED) recruited from the community into the Binge Eating Genetics Initiative Study (BEGIN) based on whether they reported ever accessing treatment or not.

BN/BED are serious and prevalent mental health illnesses affecting an estimated 1.1–3.8% of the population [4]. Both disorders involve binge eating, which is the consumption of a large amount of food accompanied by a sense of loss of control over eating. In the case of BN, individuals compensate for binge eating with behaviors such as fasting, self-induced vomiting, laxative/diuretic use, and maladaptive exercise. In BED, such compensatory behaviors do not regularly occur. BN/BED carry significant mental and physical health morbidity and are associated with elevated suicide attempt risk and functional impairment [4, 5]. Yearly, 3.3 million healthy life years are conservatively estimated to be lost worldwide from eating disorders [6]. Effective treatments are available, yet many people never access treatment [1].

Studies comparing the features of individuals with BN/BED who have accessed treatment to those who have not are uncommon. In one study, BN cases from an eating disorder clinic were compared to two groups of household-recruited cases from a population-based survey: BN cases who had never sought treatment and controls [7]. Clinic-detected cases had higher prevalences of affective and substance use disorders, followed by household-recruited cases, and then controls. Another study by Wilfley and colleagues [8] found that treatment-seeking individuals with BED had greater severity and social impairment, whereas community-recruited cases were younger, less educated, and more likely to identify as Black. In another study of people with eating disorders recruited from the community, prior treatment was associated with more functional impairment at baseline. Further, over a prospective 12-month period, higher baseline symptoms, mental health comorbidity, functional impairment, and weight differentiated those who had sought treatment from those who had not [9, 10].

Systematic reviews on help-seeking have reported that individuals who seek treatment have more severe symptoms and somatic and mental health comorbidity, which contribute to emotional distress and motivate help-seeking [11, 12]. Some studies have found that concern about weight, or higher actual body mass index (BMI) are associated with treatment access, which others have not [11, 12]. A strength of this literature is that it reflects a combination of qualitative and quantitative research findings.

Research has also highlighted sociodemographic correlates of healthcare access. The US National Epidemiological Survey reported that males and racial/ethnic minorities with eating disorders were less likely to access treatment than females and non-Hispanic Whites [13]. Traditional eating disorder healthcare was historically oriented to females under the mistaken belief that eating disorders in males were rare; today stigmatization, structural disparities, and gender-related barriers persist [14, 15]. The existing findings suggest that untreated individuals with BN/BED are likely to have milder symptoms and more diverse sociodemographic characteristics.

Studying the factors related to treatment access is important to recognize the potential sources of bias that may impact research. Clinic-based samples are overrepresented, yet three-quarters of individuals with eating disorders do not access treatment, hence, those individuals are far less likely to be represented in clinical research [1]. Additionally, studying the factors related to treatment access is critical because eating disorders can lead to significant life impairment and timely treatment can improve outcome [6]. Addressing underserved populations and identifying opportunities for improving healthcare access is essential.

This study compared individuals with BN/BED who reported never accessing treatment to those who reported having accessed treatment on sociodemographic variables and self-reported clinical symptomatology. We hypothesized that individuals who had accessed treatment would be more likely to be female, White, have more severe eating disorder and comorbid mental health and somatic symptoms, and higher weight concern and BMI, based on previous studies that have implicated these factors in treatment access [7, 8, 11, 12, 16]. To explore this further, we classified individuals by their highest level of care, and grouped treated individuals by whether they self-reported receiving residential/inpatient treatment or outpatient care. We anticipated that the untreated group would have the lowest severity and greatest sociodemographic diversity relative to the inpatient group, with the outpatient group falling somewhere in the middle.

## Methods

### Participants and procedure

This study includes participants from the Binge Eating Genetics Initiative (BEGIN) study [17] United States study arm. The BEGIN study investigates the role of genomic, gut microbiota, and behavioral factors in BN and BED. Participants were recruited from the community through social and electronic media (Twitter, Facebook, press releases, blog, listservs, a study website), the Center of Excellence for Eating Disorders volunteer research registry, university recruitment mechanisms, the National Eating Disorders Association, and the *Recovery Record* app, a self-help eating disorder mobile phone application [18]. Effort was made to recruit racially, ethnically, and gender-diverse participants (e.g., via culturally-representative blog posts and flyers). Participant recruitment took place between August 2017 and March 2021. Individuals provided informed consent and completed a HIPAA-compliant initial screening survey. The study inclusion criteria were: (1) a lifetime diagnosis of BN and/or BED based on *Diagnostic and Statistical Manual* 5th edition (DSM-5) [19] criteria and self-reported current binge-eating behavior, (2) 18–45 years, (3) U.S. resident, (4) speaks English, (5) has an iPhone version 5 or newer, (6) willing to wear an Apple Watch, and (7) willing to use the Recovery Record app over the 30-day course of the study. Individuals were excluded if they met any of the following criteria: (1) currently pregnant or breastfeeding, (2) current hormone therapy, (3) antibiotic or probiotic use within 30 days before enrolment, (4) current inpatient treatment, (5) history of bariatric surgery, or (6) current suicidality. Some exclusion criteria (i.e., antibiotic and probiotic use) were oriented toward other aspects of the study (i.e., microbiome study).

Participants from BEGIN were included in the present study if they were currently symptomatic with BN or BED, completed questions on treatment history, and had a BMI > 18.5 kg/m^2^. Current BN symptomatology was defined as a DSM-5 BN lifetime diagnosis by algorithm from the Eating Disorders 100,000 Questionnaire [20] and at least four binge-eating episodes and at least four episodes of fasting, vomiting, laxative use, or driven exercise for weight control purposes over the previous 28 days on the Eating Disorder Examination Questionnaire (EDE-Q) [21, 22]. Current BED symptomatology was defined as a DSM-5 BED lifetime diagnosis by algorithm from the ED100K.v2 and at least four binge-eating episodes and no episodes of fasting, vomiting, laxatives or driven exercise over the previous 28 days on the EDE-Q. These thresholds align with DSM-5 frequency criteria for BN and BED [19]. Subthreshold or other specified feeding and eating disorders (OSFED) similar to BN/BED were not included because of concerns regarding their reliable operationalization.

Participants completed survey measures through the *Recovery Record* app. The study was approved by the University of North Carolina Biomedical Institutional Review Board and is registered at ClinicalTrials.gov: NCT04162574.

### Measures

#### Eating disorders

Participants completed the Eating Disorders 100,000 Questionnaire version 2 (ED100K.v2), a self-report diagnostic questionnaire for lifetime eating disorders based on the Structured Clinical Interview for DSM-5 (SCID) [20]. The ED100K.v2 yields lifetime DSM-5 diagnoses of eating disorders (anorexia nervosa [AN], BN, BED) and does not assess current eating disorder diagnosis. A computer-based algorithm is applied to each participant’s completed questionnaire responses to identify lifetime diagnoses in accordance with DSM criteria. This measure was created by the Anorexia Nervosa Genetics Initiative (ANGI) for rapid, cost-effective recruitment of tens of thousands of eating disorder cases for genomics research. Module H questions from the SCID were adapted to a self-report format. High correlations (ranging from 0.91 to 0.92 across countries) were observed between the ED100K (v1) and SCID interview responses for the lowest illness-related BMI for AN diagnosis. Additionally, there was strong support for ED100K-assessed AN criterion B (positive predictive values between 0.96–1.00 across countries), AN criterion C (between 0.98 and 1.00 across countries), and binge eating (between 0.77 and 0.93 across countries) when compared to clinician-administered SCID interviews, indicating preliminary validation support for the ED100K [20].

#### Treatment history

Treatment history for BN/BED was collected using a series of questions compiled for this study to capture the most likely treatments individuals in the community would have received from primary or specialist care providers. The gateway question was “Have you ever received any of the following treatments for binge-eating disorder or bulimia nervosa? Check all that apply” followed by the options: “inpatient treatment”, “residential treatment”, “emergency room treatment”, “cognitive-behavioral therapy (individual or group)”, “interpersonal psychotherapy (individual or group)”, “other type of psychotherapy”, “I have never received any outpatient treatment for binge-eating disorder or bulimia nervosa” and “Don’t know/refuse”.

Use of psychiatric medication was assessed in the following way. Participants were presented with the question, “Have you ever received any of the following medications for binge eating? Check all that apply” followed by a list of prescription medications used in the treatment of BN and BED that included both chemical and brand names of drugs. The list included selective serotonin reuptake inhibitors (i.e., Prozac, Luvox, Zoloft, etc.), lisdexamfetamine (Vyvanse), topiramate (Topamax), bupropion (Wellbutrin), and duloxetine (Irenka, Cymbalta). Participants could also check “other medication” and provide the name in free-text. Weight loss agents such as phentermine (Adipex), orlistat (Alli), phentermine/topiramate (Qsymia), naltrexone/bupropion (Contrave), and lorcaserin (Belviq) were captured but were not considered psychiatric medications. Following data collection, the responses were reviewed for each participant and each participant was given a code of “yes” or “no” for psychiatric medication.

Participants were classified into groups based on whether they had received treatment. Participants who responded “yes” to having received any of the following were classified as “treated”: inpatient, residential, cognitive-behavioral therapy (CBT), interpersonal psychotherapy (IPT), other type of psychotherapy, and psychiatric medication. All other participants who responded, “I have never received any outpatient treatment for binge-eating disorder or bulimia nervosa”, had received only emergency room treatment, or had never received medication for binge eating were classified as “untreated.” Importantly, these groups measure having accessed treatment and are unable to address the adequacy or completion of treatment. Since the groups are based on ever having accessed treatment, treatment could have been accessed either for a past episode or during the present symptomatic episode of BN/BED.

#### Sociodemographics

Race and ethnicity were self-reported according to the National Institutes of Health (NIH) categories, along with age. Biological sex was ascertained with DNA genotyping [17]. Due to the low *n*s and small cell sizes, racial categories other than White were aggregated for analysis.

#### Eating disorder psychopathology and binge eating

The EDE-Q v 6.0 [21, 22] is a valid, reliable self-report questionnaire of cognitive and behavioral symptoms of eating disorders over the prior four weeks. The present study included the subscales (Restraint, Eating Concern, Shape Concern, Weight Concern) and Global score, which assess eating disorder psychopathology, and number of episodes of fasting, objective binge eating, self-induced vomiting, laxative use, and driven exercise. Number of fasting episodes is measured on an ordinal scale with the response options of “no days”, “1–5 days”, “6–12 days”, “13–15 days”, “16–22 days”, “23–27 days” and “every day” and the count of number of episodes of the other behaviors was measured. Higher scale scores indicate more severe symptoms.

#### Comorbid mental health symptoms

Participants completed screening measures for anxiety (Generalized Anxiety Disorder-7; GAD-7) [23], depression (Patient Health Questionnaire-9; PHQ-9) [24], and attention-deficit/hyperactivity disorder (ADHD) (Adult ADHD Self-Report Scale v1.1 Screener; ASRS-6) [25]. These yielded dimensional scores of symptom severity, with theoretical score ranges of 0–21 for GAD-7, 0–27 for PHQ-9, and 0–24 for ASRS-6. Positive screens for generalized anxiety disorder and major depression were based on a validated threshold of 10 on the GAD-7 and PHQ-9, respectively, and for screening-detected ADHD, at least four or more checkmarks in the darkly shaded boxes of the ASRS-6 [23–25]. These measures are widely used, consistent with DSM-IV diagnostic criteria, and have well-established reliability and validity [23–26]. Higher scores correspond to more severe symptoms.

#### Comorbid disorders of gut-brain interaction (DGBIs)

Since somatic comorbidity has been found to differentiate treated and untreated patients with eating disorders, the functional bowel disorders section of the Rome III diagnostic self-report questionnaire (RIIIAQ) was included [27]. The RIIIAQ was developed from Rome III diagnostic criteria [28] to assess disorders of gut-brain interaction (DGBIs), formerly called functional gastrointestinal disorders. It has good reliability and validity [27]. Irritable bowel syndrome (IBS), functional bloating, functional constipation, functional diarrhea, and unspecified functional bowel disorder were assessed, and a composite of any of these diagnoses was also created.

#### History of eating disorder behaviors

The ED100K yields a clinical history of the lifetime use, age at onset, frequency, and duration of eating disorder behaviors. The behaviors include objective binge eating, fasting, self-induced vomiting, laxatives, diuretics, diet pills, and excessive exercise. Except for binge eating, the behaviors are assessed in relation to weight and shape control, “Have you ever used any of the following to control your body shape or weight?” and then separately in relation to compensating for binge eating, “Have you ever used any of the following to compensate for episodes of binge eating or overeating”. Each question was followed by a list of the behaviors, and the respondent had the option to check all that apply or none at all. Excessive exercise was described in the list as: “Excessive exercise (e.g., feel compelled to exercise, feel uneasy or distressed if unable to exercise”. In this study, lifetime use of an eating disorder behavior was measured by reported ever-use using a binary yes/no response option. A composite representing the lifetime use of any eating disorder behavior was also created with a binary yes/no format. Age at onset was self-reported age (in years) at first use, if applicable. The ED100K assesses the duration of each behavior with the question stem of “For how long a period of time did you…” and includes six response options of “less than 1 month”, “1 to 2 months”, “3 to 5 months”, “6 to 12 months”, “more than 1 year”, and “don’t know”. Frequency of each disorder behavior was measured with the question stem “how often did you usually use…” and options of “less than once a week”, “at least once a week”, “at least twice a week”, “every day/nearly every day”, and “don’t know. For each behavior a participant did not endorse, this question was skipped with conditional branching logic. No ever-use of a behavior was added as the lowest ordinal category of “never” for the duration and frequency variables. “Don’t know” responses were treated as missing.

#### BMI history

Current, lowest, and highest weight outside of pregnancy at adult height, and adult height, were collected by self-report with the ED100K and converted to BMIs (kg/m^2^).

#### Highest level of care

The “treated” group was categorized into “inpatient” and “outpatient” groups for analyses involving highest level of care. Level of care reflects the intensity of treatment. Inpatient and residential settings provide the highest level of care, while outpatient psychotherapy or psychotropic medication management is lower in intensity. The inpatient group included any participant who self-reported inpatient and/or residential treatment for BN/BED, and the outpatient group included participants who had received outpatient and/or psychiatric medication but had never received inpatient or residential treatment. The “untreated group” was the same group described previously.

### Statistical analysis

Groups were compared on the variables in Tables 1 and 2 using models appropriate to the statistical distribution of each construct. Logistic or exact logistic regression was used to analyze the following dependent variables: biological sex, race, ethnicity, screening-detected MDD, GAD and ADHD, RIIIAQ diagnoses, and ED100K lifetime use of eating disorder behaviors. ANOVA was used to analyze the following dependent variables: age, the EDE-Q, GAD-7, PHQ-9, and ASRS-6 scale scores, age at onset, and BMI measures. Additionally, logistic ordinal regression was used to compare groups on EDE-Q fasting, and ED100K duration and frequency of eating disorder behaviors. The proportional odds assumption was evaluated and was tenable in all instances. Negative binomial regression was used for number of episodes of binge eating measured with the EDE-Q. Zero-inflated negative binomial models accommodated the excess zeros and positively skewed distributions of the EDE-Q vomiting, laxative, and driven exercise measures. We tested the models and repeated them in sensitivity analyses adjusting for a lifetime history of AN diagnosis (see Additional file 1: Table S1–S2). The false discovery rate (FDR) procedure corrected for multiple comparisonss [29]. FDR *p* values < 0.05 were interpreted as being significant, and uncorrected *p* values are reported for completenes. Prior to analysis, three variables with > 5% missingness assumed to be missing at random, were imputed with multiple imputation. The highest percentage of missing values for any variable was 15%. Multiple imputation results were combined with Rubin [30] and Schafer’s [31] methods. We conducted a sensitivity analysis using only complete cases, and the results were similar, hence the primary analysis is presented. There was a small amount of missing data on biological sex (*n* < 5), hence we suppressed sample sizes in the text and tables where cell sizes could be inferred, to comply with privacy regulations. For biological sex, percentages were based on available data. The analyses were conducted in SAS version 9.4 (SAS Inc, Cary, NC, US).

**Table 1:** Comparison of research participants with BN/BED who had never accessed treatment (Untreated) vs those who had accessed treatment (Treated)

Variable	Untreated (*n* = 291)	Treated (*n* = 471)	Difference between groups Estimate (95% CI)	*p*	FDR *p*
Sociodemographics					
Biological sex					
Female	228 (78%)	93%^a^	OR 3.43 (2.20, 5.34)	< 0.001***	< 0.001***
Male	63 (22%)	7%^a^			
Age, y	29.13 (7.43)	29.75 (7.25)	Cohen’s *d* −0.08 (−0.23, 0.06)	0.26	0.33
Race					
White	232 (83%)	418 (90%)	OR 2.01 (1.34, 3.00)	< 0.001***	0.01*
Racial minorities	49 (17%)	48 (10%)			
Ethnicity^b^					
Non-Hispanic	256 (88%)	432 (92%)	OR 1.51 (0.94, 2.45)	0.09	0.16
Hispanic	35 (12%)	39 (8%)			
Current clinical features					
Mental health					
EDE-Q restraint	3.01 (1.56)	3.01 (1.51)	Cohen’s *d* 0.00 (−0.15, 0.15)	0.95	0.97
EDE-Q eating concern	3.71 (1.16)	3.87 (1.16)	Cohen’s *d* −0.14 (−0.28, 0.01)	0.07	0.13
EDE-Q shape concern	4.78 (1.08)	4.85 (0.94)	Cohen’s *d* −0.07 (−0.22, 0.08)	0.40	0.46
EDE-Q weight concern	4.41 (1.14)	4.67 (0.94)	Cohen’s *d* −0.25 (−0.40, −0.11)	< .0.001***	0.007**
EDE-Q global	3.98 (0.96)	4.10 (0.87)	Cohen’s *d* −0.13 (−0.28, 0.01)	0.08	0.16
EDE-Q binge eating	14.22 (8.07)	14.92 (10.45)	IRR 1.05 (0.96, 1.15)	0.28	0.35
EDE-Q fasting	1.48 (1.70)	1.39 (1.69)	OOR 0.89 (0.68, 1.15)	0.37	0.45
EDE-Q self-induced vomiting^c^	0 (0, 0)	0 (0, 2)	OR 0.54 (0.36, 0.82) IRR 1.64 (1.07, 2.52)	0.003** 0.02*	0.02* 0.08
EDE-Q laxatives^c^	0 (0, 0)	0 (0, 0)	OR 1.09 (0.69, 1.74) IRR 0.86 (0.51, 1.45)	0.71 0.58	0.77 0.64
EDE-Q driven exercise^c^	0 (0, 8)	1 (0, 5)	OR 0.96 (0.70, 1.32) IRR 0.86 (0.71, 1.04)	0.82 0.13	0.87 0.20
PHQ-9 score	11.58 (5.38)	12.53 (5.08)	Cohen’s *d* −0.18 (−0.33, −0.03)	0.02*	0.08
GAD-7 score	9.37 (5.57)	10.20 (5.22)	Cohen’s *d* −0.15 (−0.31, −0.01)	0.04*	0.10
ASRS-6 score	11.38 (5.60)	12.75 (5.48)	Cohen’s *d* −0.25 (−0.40, −0.10)	0.001**	0.009**
Screening-detected MDD (PHQ-9)	169 (58%)	321 (68%)	OR 1.54 (1.14, 2.09)	0.005**	0.02*
Screening-detected GAD (GAD-7)	123 (46%)	243 (54%)	OR 1.38 (1.02, 1.87)	0.04*	0.09
Screening-detected ADHD (ASRS-6)	126 (47%)	248 (55%)	OR 1.38 (1.02, 1.87)	0.04*	0.09
DGBIs					
IBS	76 (28%)	164 (36%)	OR 1.45 (1.04, 2.01)	0.03*	0.08
Functional bloating	74 (28%)	124 (27%)	OR 1.00 (0.71, 1.40)	0.98	0.98
Functional constipation	21 (8%)	20 (4%)	OR 0.55 (0.29, 1.03)	0.06	0.13
Functional diarrhoea	^d^	^d^	OR 2.41 (0.51, 11.41)	0.27	0.34
Unspecified functional bowel disorder	185 (69%)	336 (74%)	OR 1.32 (0.94, 1.84)	0.11	0.17
Any diagnosis above	225 (84%)	389 (86%)	OR 1.21 (0.79, 1.84)	0.38	0.45
BMI kg/m^2^	31.66 (8.29)	33.29 (10.31)	Cohen’s *d* −0.17 (−0.32, −0.02)	0.02*	0.08
Lifetime clinical history					
Eating disorder behavior					
Age at onset of binge eating	18.88 (7.08)	17.19 (6.45)	Cohen’s *d* 0.25 (0.09, 0.41)	0.02*	0.08
Age at onset of eating disorder behavior	17.06 (6.17)	14.90 (5.10)	Cohen’s *d* 0.39 (0.24, 0.54)	< .0.001***	< 0.001***
Prevalence of behaviors to control weight or shape					
Vomiting	132 (45%)	288 (61%)	OR 1.90 (1.41, 2.55)	< .0.001***	< .0.001***
Laxatives	102 (35%)	201 (43%)	OR 1.38 (1.02, 1.87)	0.04*	0.09
Diuretics	70 (24%)	139 (30%)	OR 1.32 (0.95, 1.85)	0.10	0.17
Diet pills	153 (53%)	274 (58%)	OR 1.25 (0.93, 1.68)	0.13	0.20
Exercising excessively	212 (73%)	376 (80%)	OR 1.47 (1.05, 2.08)	0.03*	0.08
Fasting	232 (80%)	378 (80%)	OR 1.03 (0.72, 1.49)	0.86	0.89
Overall (any of the above)	267 (92%)	449 (95%)	OR 1.83 (1.01, 3.34)	0.04*	0.10
Prevalence of behaviors to compensate for binge eating or overeating					
Vomiting	105 (36%)	265 (56%)	OR 2.28 (1.69, 3.08)	< .0.001***	< .0.001***
Laxatives	82 (28%)	186 (39%)	OR 1.66 (1.21, 2.28)	0.002**	0.01**
Diuretics	45 (15%)	91 (19%)	OR 1.31 (0.88, 1.94)	0.18	0.25
Diet pills	115 (40%)	212 (45%)	OR 1.25 (0.93, 1.69)	0.14	0.20
Exercising excessively	182 (63%)	309 (66%)	OR 1.14 (0.84, 1.55)	0.39	0.46
Fasting	212 (73%)	330 (70%)	OR 0.87 (0.63, 1.21)	0.41	0.46
Overall (any of the above)	255 (88%)	428 (91%)	OR 1.41 (0.88, 2.25)	0.16	0.22
Symptom duration					
Binge eating					
Less than 1 month	^d^	^d^	OOR 1.78 (1.29, 2.45)	< .0.001***	0.005**
1 to 2 months	^d^	^d^			
3 to 5 months	38 (14%)	39 (8%)			
6 to 12 months	49 (18%)	62 (13%)			
More than 1 year	175 (64%)	350 (76%)			
Vomiting					
Never	159 (57%)	183 (40%)	OOR 1.39 (1.01, 1.92)	0.04*	0.10
Less than 1 month	45 (16%)	56 (12%)			
1 to 2 months	21 (7%)	27 (6%)			
3 to 5 months	14 (5%)	25 (5%)			
6 to 12 months	16 (6%)	37 (8%)			
More than 1 year	26 (9%)	129 (28%)			
Laxatives					
Never	189 (67%)	270 (59%)	OOR 1.32 (0.95, 1.84)	0.10	0.17
Less than 1 month	35 (12%)	50 (11%)			
1 to 2 months	22 (8%)	32 (7%)			
3 to 5 months	12 (4%)	33 (7%)			
6 to 12 months	7 (2%)	21 (5%)			
More than 1 year	18 (6%)	48 (11%)			
Diuretics					
Never	221 (79%)	332 (72%)	OOR 1.39 (0.95, 2.02)	0.09	0.16
Less than 1 month	21 (8%)	44 (10%)			
1 to 2 months	13 (5%)	25 (5%)			
3 to 5 months	10 (4%)	17 (4%)			
6 to 12 months	5 (2%)	16 (3%)			
More than 1 year	8 (3%)	24 (5%)			
Diet pills					
Never	138 (48%)	197 (43%)	OOR 1.28 (0.95, 1.74)	0.10	0.17
< 1 month	34 (12%)	47 (10%)			
1 to 2 months	30 (10%)	56 (12%)			
3 to 5 months	17 (6%)	48 (10%)			
6 to 12 months	28 (10%)	45 (10%)			
More than 1 year	39 (14%)	68 (15%)			
Exercising excessively					
Never	79 (29%)	95 (22%)	OOR 1.41 (0.99, 2.02)	0.06	0.12
Less than 1 month	16 (6%)	21 (5%)			
1 to 2 months	18 (7%)	18 (4%)			
3 to 5 months	21 (8%)	31 (7%)			
6 to 12 months	30 (11%)	63 (15%)			
More than 1 year	107 (39%)	199 (47%)			
Fasting					
Never	59 (21%)	93 (21%)	OOR 1.25 (0.90, 1.74)	0.18	0.25
Less than 1 month	60 (22%)	69 (15%)			
1 to 2 months	26 (9%)	40 (9%)			
3 to 5 months	18 (7%)	29 (6%)			
6 to 12 months	24 (9%)	58 (13%)			
More than 1 year	88 (32%)	157 (35%)			
Symptom frequency					
Binge eating					
Less than once per week	8 (3%)	10 (2%)	OOR 1.38 (1.04, 1.85)	0.03*	0.08
At least once a week	8 (3%)	19 (4%)			
At least twice a week	138 (49%)	179 (39%)			
Every day or nearly every day	129 (46%)	254 (55%)			
Vomiting					
Never	159 (56%)	183 (39%)	OOR 2.26 (1.70, 2.99)	< .0.001***	< .0.001***
Less than once per week	74 (26%)	108 (23%)			
At least once a week	18 (6%)	39 (8%)			
At least twice a week	14 (5%)	44 (9%)			
Every day or nearly every day	18 (6%)	90 (19%)			
Laxatives					
Never	189 (67%)	270 (59%)	OOR 1.38 (1.02, 1.86)	0.04*	0.09
Less than once per week	46 (16%)	88 (19%)			
At least once a week	23 (8%)	37 (8%)			
At least twice a week	13 (5%)	26 (6%)			
Every day or nearly every day	12 (4%)	33 (7%)			
Diuretics					
Never	221 (79%)	332 (72%)	OOR 1.39 (0.98, 1.87)	0.06	0.13
Less than once per week	29 (10%)	62 (14%)			
At least once a week	12 (4%)	20 (4%)			
At least twice a week	7 (2%)	18 (4%)			
Every day or nearly every day	12 (4%)	26 (6%)			
Diet pills					
Never	138 (49%)	197 (43%)	OOR 1.21 (0.91, 1.59)	0.19	0.25
Less than once per week	18 (6%)	43 (9%)			
At least once a week	11 (4%)	26 (6%)			
At least twice a week	16 (6%)	20 (4%)			
Every day or nearly every day	98 (35%)	175 (38%)			
Exercising excessively					
Never	79 (28%)	95 (21%)	OOR 1.49 (1.11, 1.98)	0.007**	0.03*
Less than once per week	^d^	7 (2%)			
At least once a week	^d^	10 (3%)			
At least twice a week	43 (15%)	64 (14%)			
Every day or nearly every day	140 (50%)	266 (60%)			
Fasting					
Never	59 (22%)	93 (21%)	OOR 1.03 (0.79, 1.34)	0.85	0.89
Less than once per week	45 (16%)	74 (16%)			
At least once a week	51 (19%)	74 (16%)			
At least twice a week	54 (20%)	114 (25%)			
Every day or nearly every day	65 (24%)	96 (21%)			
BMI history					
Lowest adult BMI kg/m^2^	23.82 (5.31)	23.30 (6.04)	Cohen’s *d* 0.09 (−0.06, 0.24)	0.22	0.29
Highest adult BMI kg/m^2^	34.25 (8.41)	36.47 (10.66)	Cohen’s *d* −0.22 (−0.37, −0.08)	0.003**	0.02*

Values are *n* (%) or *M* (*SD*) unless stated otherwise. Percentages are based on non-missing data. ASRS-6 = Adult ADHD Self-Report Scale v1.1 Screener; DGBIs = disorders of gut-brain interaction; EDE-Q = Eating Disorder Examination-Questionnaire; FDR = false discovery rate. GAD = generalized anxiety disorder; GAD-7 = Generalized Anxiety Disorder-7; IRR = incidence rate ratio; MDD = major depressive disorder; OOR = ordinal odds ratio; OR = odds ratio; PHQ-9 = Patient Health Questionnaire9

^a^*n*s are suppressed to protect the privacy of participants with missing data, see the Statistical Analysis section for further information

^b^Post hoc analyses among females showed significant differences between the untreated and treated groups (see the text)

^c^Because of non-normal, excess zero, positively skewed distributions, median and IQR are given. The ORs are the parameter estimates for the ‘excess zero’ part, and the IRRs are the parameter values for the ‘count’ part of the zero-inflated regression model. The upper *p* is for the OR and the lower *p* is for the IRR

^d^Confidentiality of individually identifiable information was maintained by suppressing *n*s and percents for cells with fewer than 5 participants, as well as adjacent or complimentary cells that could lead to recalculation of suppressed cells

*p < 0.05, **p < 0.01, ***p < 0.001

**Table 2:** Comparison of research participants with BN/BED by highest level of care received for BN/BED

Variable	Inpatient/residential (*n* = 86)	Outpatient or Medication (*n* = 385)	Untreated (*n* = 291)	*p*	FDR *p*	Difference between groups
Sociodemographics						
Biological sex						
Female	95%^a^	92%^a^	228 (78%)	< 0.001***	< 0.001***	Untreated > inpatient & outpatient
Male	5%^a^	8%^a^	63 (22%)			
Age, y	28.45 (7.32)	30.04 (7.22)	29.13 (7.43)	0.10	0.16	–
Race						
White	80 (93%)	338 (89%)	232 (83%)	0.002**	0.009**	Untreated > inpatient & outpatient
Racial minorities	6 (7%)	42 (11%)	49 (17%)			
Ethnicity						
Non-Hispanic	^b^	^b^	256 (88%)	0.12	0.18	–
Hispanic	^b^	^b^	35 (12%)			
Current clinical features						
Mental health						
EDE-Q restraint	3.24 (1.38)	2.95 (1.54)	3.01 (1.56)	0.29	0.35	–
EDE-Q eating concern	3.96 (1.11)	3.85 (1.18)	3.71 (1.16)	0.14	0.20	–
EDE-Q shape concern	4.81 (0.87)	4.86 (0.96)	4.78 (1.08)	0.65	0.68	–
EDE-Q weight concern	4.69 (0.95)	4.66 (0.94)	4.41 (1.14)	0.004**	0.01*	Inpatient & outpatient > untreated
EDE-Q global	4.18 (0.82)	4.08 (0.88)	3.98 (0.96)	0.15	0.21	–
EDE-Q binge eating	15.20 (11.44)	14.86 (10.23)	14.22 (8.07)	0.53	0.57	–
EDE-Q fasting	1.55 (1.66)	1.36 (1.70)	1.48 (1.70)	0.30	0.36	–
EDE-Q self-induced vomiting^c^	0 (2, 14)	0 (0, 1)	0 (0, 0)	< 0.001*** < 0.001***	< 0.001*** 0.003**	Inpatient > outpatient & untreated Outpatient > untreated
EDE-Q laxatives^c^	0 (0, 0)	0 (0, 0)	0 (0, 0)	0.19 0.85	0.26 0.87	–
EDE-Q driven exercise^c^	0 (0, 10)	0 (0, 5)	0 N	0.005** 0.32	0.01* 0.37	Inpatient > outpatient & untreated
PHQ-9 score	13.60 (5.42)	12.29 (4.97)	11.58 (5.38)	0.007**	0.02*	Inpatient > untreated
GAD-7 score	11.45 (4.92)	9.92 (5.25)	9.37 (5.57)	0.008**	0.02*	Inpatient > outpatient & untreated
ASRS-6 score	11.45 (4.92)	9.92 (5.25)	11.38 (5.60)	0.001**	0.006**	Inpatient & outpatient > untreated
Screening-detected MDD (PHQ-9)	66 (77%)	130 (34%)	169 (58%)	0.004**	0.01*	Inpatient & outpatient > untreated
Screening-detected GAD (GAD-7)	51 (61%)	192 (52%)	123 (46%)	0.03*	0.08	–
Screening-detected ADHD (ASRS-6)	52 (63%)	196 (53%)	126 (47%)	0.03*	0.08	–
DGBIs						
IBS	34 (41%)	130 (35%)	76 (28%)	0.05	0.10	–
Functional bloating	22 (27%)	102 (28%)	74 (28%)	0.98	0.98	–
Functional constipation	5 (6%)	15 (4%)	21 (8%)	0.14	0.20	–
Functional diarrhoea	^b^	^b^	^b^	0.43	0.48	–
Unspecified functional bowel disorder	68 (82%)	268 (74%)	185 (69%)	0.07	0.12	–
Any diagnosis above	76 (92%)	313 (85%)	225 (84%)	0.21	0.28	–
BMI kg/m^2^	31.19 (11.16)	33.76 (10.06)	31.66 (8.29)	0.006*	0.02*	Outpatient > inpatient & untreated
Lifetime clinical history						
Eating disorder behavior						
Age at onset of binge eating	16.23 (6.23)	17.42 (6.49)	18.88 (7.08)	0.003**	0.01*	Inpatient & outpatient < untreated
Age at onset of eating disorder behavior	13.49 (3.85)	15.22 (5.30)	17.06 (6.17)	< 0.001***	< 0.001***	Inpatient < outpatient < untreated
Prevalence of behaviors to control weight or shape						
Vomiting	69 (80%)	219 (57%)	132 (45%)	< 0.001***	< 0.001***	Inpatient > outpatient > untreated
Laxatives	45 (52%)	156 (41%)	102 (35%)	0.02*	0.04*	Inpatient > untreated
Diuretics	31 (36%)	108 (28%)	70 (24%)	0.09	0.15	–
Diet pills	48 (56%)	226 (59%)	153 (53%)	0.28	0.35	–
Exercising excessively	76 (88%)	300 (78%)	212 (73%)	0.01*	0.03*	Inpatient > outpatient & untreated
Fasting	73 (85%)	305 (79%)	232 (80%)	0.49	0.53	–
Overall (any of the above)	85 (99%)	364 (95%)	267 (92%)	0.07	0.13	–
Prevalence of behaviors to compensate for binge eating or overeating						
Vomiting	69 (80%)	196 (51%)	105 (36%)	< 0.001***	< 0.001***	Inpatient > outpatient > untreated
Laxatives	40 (47%)	146 (38%)	82 (28%)	0.002**	0.01*	Inpatient & outpatient > untreated
Diuretics	23 (27%)	68 (18%)	45 (15%)	0.06	0.11	–
Diet pills	39 (45%)	173 (45%)	115 (40%)	0.33	0.37	–
Exercising excessively	64 (74%)	245 (64%)	182 (63%)	0.12	0.18	–
Fasting	67 (78%)	263 (68%)	212 (73%)	0.15	0.21	–
Overall (any of the above)	84 (98%)	344 (89%)	255 (88%)	0.05	0.10	–
Symptom duration						
Binge eating						
Less than 1 month	^b^	^b^	^b^	< 0.001***	0.002**	Inpatient > outpatient > untreated
1 to 2 months	^b^	^b^	^b^			
3 to 5 months	5 (5%)	34 (9%)	38 (14%)			
6 to 12 months	7 (8%)	55 (15%)	49 (18%)			
More than 1 year	74 (86%)	276 (74%)	175 (64%)			
Vomiting						
Never	17 (20%)	166 (44%)	159 (57%)	0.02*	0.06	–
Less than 1 month	^b^	^b^	45 (16%)			
1 to 2 months	^b^	^b^	21 (7%)			
3 to 5 months	^b^	^b^	14 (5%)			
6 to 12 months	8 (10%)	29 (8%)	16 (6%)			
More than 1 year	47 (56%)	82 (22%)	26 (9%)			
Laxatives						
Never	41 (48%)	229 (62%)	189 (67%)	0.09	0.15	–
Less than 1 month	7 (8%)	43 (12%)	35 (12%)			
1 to 2 months	6 (7%)	26 (7%)	22 (8%)			
3 to 5 months	7 (6%)	26 (7%)	12 (4%)			
6 to 12 months	6 (7%)	15 (4%)	7 (2%)			
More than 1 year	18 (21%)	30 (8%)	18 (6%)			
Diuretics						
Never	55 (65%)	277 (74%)	221 (79%)	0.09	0.16	–
Less than 1 month	9 (11%)	35 (9%)	21 (8%)			
1 to 2 months	^b^	^b^	13 (5%)			
3 to 5 months	^b^	^b^	10 (4%)			
6 to 12 months	5 (6%)	11 (3%)	5 (2%)			
More than 1 year	8 (9%)	16 (4%)	8 (3%)			
Diet pills						
Never	38 (45%)	159 (42%)	138 (48%)	0.27	0.35	–
Less than 1 month	6 (7%)	41 (11%)	34 (12%)			
1 to 2 months	8 (9%)	48 (13%)	30 (10%)			
3 to 5 months	11 (13%)	37 (10%)	17 (6%)			
6 to 12 months	9 (11%)	36 (10%)	28 (10%)			
More than 1 year	13 (15%)	55 (15%)	39 (14%)			
Exercising excessively						
Never	10 (12%)	85 (24%)	79 (29%)	0.04*	0.10	–
Less than 1 month	^b^	^b^	16 (6%)			
1 to 2 months	^b^	^b^	18 (7%)			
3 to 5 months	9 (11%)	22 (6%)	21 (8%)			
6 to 12 months	9 (11%)	54 (16%)	30 (11%)			
More than 1 year	47 (59%)	152 (44%)	107 (39%)			
Fasting						
Never	13 (16%)	80 (22%)	59 (21%)	0.28	0.35	–
Less than 1 month	5 (6%)	64 (18%)	60 (22%)			
1 to 2 months	^b^	^b^	26 (9%)			
3 to 5 months	^b^	^b^	18 (7%)			
6 to 12 months	11 (14%)	47 (13%)	24 (9%)			
More than 1 year	45 (56%)	112 (31%)	88 (32%)			
Symptom frequency						
Binge eating						
Less than once per week	^b^	^b^	8 (3%)	0.001**	0.006**	Inpatient > outpatient > untreated
At least once a week	^b^	^b^	8 (3%)			
At least twice a week	^b^	^b^	138 (49%)			
Every day or nearly every day	59 (69%)	195 (52%)	129 (46%)			
Vomiting						
Never	17 (20%)	166 (44%)	159 (56%)	< 0.001***	< 0.001***	Inpatient > outpatient > untreated
Less than once per week	14 (16%)	94 (25%)	74 (26%)			
At least once a week	9 (10%)	30 (8%)	18 (6%)			
At least twice a week	16 (19%)	28 (7%)	14 (5%)			
Every day or nearly every day	30 (35%)	60 (16%)	18 (6%)			
Laxatives						
Never	41 (50%)	229 (62%)	189 (67%)	0.008**	0.02*	Inpatient > outpatient & untreated
Less than once per week	15 (18%)	73 (20%)	46 (16%)			
At least once a week	12 (15%)	25 (7%)	23 (8%)			
At least twice a week	5 (6%)	21 (6%)	13 (5%)			
Every day or nearly every day	9 (11%)	24 (6%)	12 (4%)			
Diuretics						
Never	55 (65%)	277 (74%)	221 (79%)	0.03*	0.06	–
Less than once per week	13 (15%)	49 (13%)	29 (10%)			
At least once a week	^b^	^b^	12 (4%)			
At least twice a week	^b^	^b^	7 (2%)			
Every day or nearly every day	6 (7%)	20 (5%)	12 (4%)			
Diet pills						
Never	38 (46%)	159 (42%)	138 (49%)	0.32	0.37	–
Less than once per week	9 (11%)	34 (9%)	18 (6%)			
At least once a week	^b^	^b^	11 (4%)			
At least twice a week	^b^	^b^	16 (6%)			
Every day or nearly every day	29 (35%)	146 (39%)	98 (35%)			
Exercising excessively						
Never	^b^	^b^	79 (28%)	0.001***	0.006**	Inpatient > outpatient & untreated
Less than once per week	^b^	^b^	^b^			
At least once a week	^b^	^b^	^b^			
At least twice a week	11 (13%)	53 (15%)	43 (15%)			
Every day or nearly every day	60 (72%)	206 (57%)	140 (50%)			
Fasting						
Never	13 (16%)	80 (22%)	59 (22%)	0.65	0.68	–
Less than once per week	11 (13%)	63 (17%)	45 (16%)			
At least once a week	18 (22%)	56 (15%)	51 (19%)			
At least twice a week	24 (29%)	90 (24%)	54 (20%)			
Every day or nearly every day	17 (20%)	79 (21%)	65 (24%)			
BMI history						
Lowest adult BMI kg/m^2^	20.76 (6.08)	23.86 (5.89)	23.82 (5.31)	< 0.001***	< .001***	Inpatient < outpatient & untreated
Highest adult BMI kg/m^2^	34.74 (11.34)	36.85 (10.48)	34.25 (8.41)	0.002**	0.01*	Outpatient > untreated

Values are *n* (%) or *M* (*SD*) unless stated otherwise. Percentages are based on non-missing data. ASRS-6 = Adult ADHD Self-Report Scale v1.1 Screener; DGBIs = disorders of gut-brain interaction; EDE-Q = Eating Disorder Examination-Questionnaire; FDR = false discovery rate. GAD = generalized anxiety disorder; GAD-7 = Generalized Anxiety Disorder-7; MDD = major depressive disorder; PHQ-9 = Patient Health Questionnaire9

^a^*n*s are suppressed to protect the privacy of participants with missing data, see the Statistical Analysis section for further information

^b^Confidentiality of individually identifiable information was maintained by suppressing *n*s and percents for cells with fewer than 5 participants, as well as adjacent or complimentary cells that could lead to recalculation of suppressed cells

^c^Because of non-normal, excess zero, positively skewed distributions, median and IQR are given. The upper *p* is for the OR of the ‘excess zero’ part and the lower *p* is for the IRR of the ‘count’ part of the zero-inflated regression model

^*^p < 0.05. **p < 0.01. ***p < 0.001

## Results

### Sample characteristics

There were 762 participants in the full sample, which included 576 individuals with current BN symptomatology and 186 with current BED symptomatology. Biological sex at birth determined by genotype was mostly female (87%), otherwise male (13%). Race was self-reported as 87% White, 6% mixed race, 3% Asian, 3% Black/African American, and 1% Native American. Ethnicity was reported as 90% non-Hispanic and 10% Hispanic. The mean ± SD for age was 30 ± 7 years. The mean number of binge-eating episodes over the past 28 days was 14.65 ± 9.61 and the mean global EDE-Q score was 4.05 ± 0.91, which are comparable to other acute samples in the literature [32, 33].

### Treatment history

The sample was divided into two groups: a “treated group” (*n* = 471) and an “untreated group” (*n* = 291) based on self-reported treatment use. Approximately 62% of the sample self-reported treatment for BN/BED at some point in their life. From most to least common, the treatments included psychiatric medication (45%, *n* = 341), CBT (39%, *n* = 294), IPT (26%, *n* = 196), inpatient care (8%, *n* = 59), residential care (8%, *n* = 58), and other psychotherapy (4%, *n* = 28). Some reported multiple treatments. Approximately 11% reported inpatient or residential care as their highest level of care (“inpatient group” *n* = 86), and 51% reported outpatient care (i.e., CBT, IPT, other psychotherapy, psychiatric medication) (“outpatient group” *n* = 385). Emergency room care (4%) and weight-loss medication (9%) are given here for descriptive purposes but were not used for classification into the treated group.

The untreated group included 214 (74%) individuals with BN and 77 (26%) with BED, and the treated group included 362 (77%) with BN and 109 (23%) with BED, with no significant difference in proportions, $\chi$^2^(1) = 1.07, *p* = 0.30. Individuals with a lifetime diagnosis of AN were significantly more likely to report receiving treatment for BN/BED ($\chi$^2^(1) = 9.57, *p* = 0.002). Around 18% of the treated group and 10% of the untreated group had a history of AN, which was 15% of the sample. Because of this finding, sensitivity analyses were conducted by adding a history of AN as a covariate to the models to provide more context for interpreting the findings (see Additional file 1: Table S1-S2). The primary results are discussed by default, and differences are pointed out where applicable.

### Sociodemographics

The untreated group had a significantly higher proportion of males and racial minorities than the treated group (Table 1). There were no significant differences on age or proportion of Hispanic participants. Although, a post hoc test among females indicated that Hispanic females were more likely to be untreated (45%) than non-Hispanic females (33%), $\chi$^2^(1) = 3.94, *p* = 0.04 and less likely to report inpatient care (Fisher’s exact test *p* = 0.03). A post hoc test among males was not carried out because of low numbers.

There were significant differences by level of care on sex and race (FDR *p*s < 0.05). Compared with females, males were > 3 times more likely to report being untreated than to report inpatient (OR = 5.65, 95% CI = 2.00, 16.13, FDR *p* = 0.004) or outpatient care (OR = 3.13, 95% CI = 1.98, 4.98, FDR *p* < 0.001). Compared with White participants, racial minorities were more likely to report being untreated than to report inpatient (OR = 3.39, 95% CI = 1.41, 8.13, FDR *p* = 0.01) or outpatient care (OR = 1.83, 95% CI = 1.20, 2.78, FDR *p* = 0.01). There were no significant differences in ethnicity or age across levels of care.

### Current clinical features

#### Eating disorder psychopathology and behaviors

The untreated group had less severe current eating disorder symptoms, particularly compared to the inpatient group. EDE-Q weight concern was significantly higher in the treated group than the untreated group (FDR *p* = 0.007; Table 1). Higher current BMI accounted for part of the variability in higher weight concern (*p* < 0.001), but the group difference remained (*p* = 0.007). Weight concern was significantly higher in both the inpatient and outpatient groups relative to the untreated group (FDR *p*s < 0.05). The other EDE-Q subscales and global score were not significantly different between the groups. The treated group had a higher severity of vomiting and driven exercise symptoms. Compared with the untreated group, the inpatient group had 11 times higher odds of vomiting (OR = 11.17, 95% CI = 3.37, 37.05, FDR *p* < 0.001) and 2 times higher odds of driven exercise (OR = 2.19, 95% CI = 1.22, 3.91, FDR *p* = 0.02), Moreover, among those with positive counts of vomiting episodes, the outpatient group had more frequent vomiting than the untreated group (IRR = 1.80, 95% CI = 1.06, 3.03, FDR *p* = 0.04). The inpatient group was more likely than the outpatient group to engage in vomiting (OR = 8.74, 95% CI = 2.67, 28.59, FDR *p* = 0.001) and driven exercise (OR = 2.46, 95% CI = 1.39, 4.34, FDR *p* < 0.001). These differences on driven exercise were not significant in the model adjusting for a history of AN, suggesting that exercise for weight control was especially prevalent in participants with a history of AN (Additional file 1: Table S2). There were no significant differences between the treated and untreated groups or the level of care groups in the odds of engaging in binge eating, fasting, or taking laxatives, compared to not engaging in these behaviors, or in the frequency of these behaviors among those who did engage in them (FDR *p*s < 0.05).

#### Comorbid mental health symptoms

Mental health symptoms were prominent in the sample, with average scores corresponding to moderate depression symptoms (PHQ-9: *M* = 12.18, *SD* = 5.21) and mild-moderate anxiety symptoms (GAD-7: *M* = 9.89, *SD* = 5.37) according to the widely used PHQ-9 and GAD-7 thresholds. Additionally, 64%, 48%, and 49% of the overall sample met MDD, GAD, and ADHD screening criteria, respectively [23, 24]. In general, the untreated group had significantly milder comorbid mental health symptoms. As shown in Table 1, the untreated group had lower depression, anxiety, and ADHD symptoms, and were less likely to screen positive for MDD, GAD, and ADHD, compared with the treated group in the uncorrected models; but after multiple testing correction, only ADHD symptom severity and screening positive for MDD were significantly different. The level of care analyses showed that as level of care increased, severity of comorbid mental health symptoms increased. Both the inpatient and outpatient groups had significantly greater levels of depression and ADHD symptoms than the untreated group. Further, the inpatient group had greater anxiety symptoms, which may have been accounted for or at least was most commonly observed among those with a history of AN.

#### Comorbid DGBIs

DGBIs were common at the time of assessment, with 85% of the sample screening positive for any type of DGBI assessed. Unspecified functional bowel disorder (68%), IBS (31%), functional bloating (26%), and functional constipation (5%) were all relatively common. There were no significant differences between the untreated and treated groups on the prevalence of any of these diagnoses (FDR *p*s > 0.05). Uncorrected *p* showed a trend for a lower prevalence of unspecified functional bowel disorder in the untreated group (Table 2).

#### Current BMI

The mean current BMI of the sample was 32.67 kg/m^2^ (*SD* = 9.61). Untreated participants had a significantly lower BMI than treated participants, corresponding to a 1.6 kg/m^2^ difference. The untreated group had a significantly lower BMI than the outpatient group, corresponding to a 2.1 kg/m^2^ difference.

### Lifetime clinical history

#### History of eating disorder behaviors

The lifetime prevalence of behaviors used to control weight and shape in the overall sample was: fasting (80%), excessive exercise (77%), diet pill use (56%), self-induced vomiting (55%), laxative use (40%), and diuretic use (27%), and only a minority (6%) reported never having used any weight control behaviors. The prevalence of behaviors to compensate for binge eating or overeating was also high. The most common was fasting (71%), and only 10% reported no use of any compensatory behavior. The mean age at onset of eating disorder behaviors in the sample was 15.71 years (*SD* = 5.60) and the mean age at onset of binge eating was somewhat higher at 17.82 years (*SD* = 6.74).

Differences were present on lifetime history of eating disorder behaviors between the untreated and treated groups. Untreated participants reported an approximately two-year later onset of eating disorder behaviors than treated participants, *F*(1, 726) = 26.15, FDR *p* < 0.001). The untreated group was the least likely to ever use weight control behaviors such as vomiting, laxatives, or excessive exercise for weight control, or compensatory behaviors. Compared with the untreated group, the inpatient group was 2–7 times more likely to report ever-use of vomiting (OR = 4.89, 95% CI = 2.74, 8.72, FDR *p* < 0.001), laxatives (OR = 2.03, 95% CI = 1.25, 3.31, FDR *p* = 0.01), and excessive exercise for weight control (OR = 2.83, 95% CI = 1.39, 5.75, FDR *p* = 0.01), and vomiting (OR = 7.19, 95% CI = 4.02, 12.87, FDR *p* < 0.001) and laxative use (OR = 2.22, 95% CI = 1.35, 3.63, FDR *p* = 0.005) to compensate for binge eating. The outpatient group was 1.5–1.8 times more likely to report vomiting for weight control (OR = 1.59, 95% CI = 1.17, 2.16, FDR *p* = 0.008) and vomiting (OR = 1.84, 95% CI = 1.35, 2.51, FDR *p* < 0.001) and laxative use to compensate for binge eating (OR = 1.56, 95% CI = 1.12, 2.16, FDR *p* = 0.02) compared with the treated group. Some of these differences attenuated when adjusting for lifetime history of AN (Additional file 1: Table S2).

Binge-eating duration was longer than a year for 69% of the sample. The duration was longer in the inpatient (ordinal odds ratio (OOR) = 3.46, 95% CI = 1.79, 6.66, FDR *p* = 0.001) and outpatient groups (OOR = 1.57, 95% CI = 1.13, 2.20, FDR *p* = 0.02) than the untreated group, and the inpatient group had a longer duration than the untreated group (OOR = 2.19, 95% CI = 1.14, 4.20, FDR *p* = 0.03). There were no significant differences between any groups in the duration of vomiting, laxative, diuretic, diet pill, excessive exercise or fasting symptoms (FDR *ps* > 0.05). There were several differences on symptom frequency. A higher frequency of symptoms such as binge eating, vomiting, laxatives, and excessive exercise was observed in the inpatient group, followed generally by the outpatient group, followed lastly by the untreated group (pairwise FDR *p*s < 0.05). In comparisons between the untreated and the inpatient group, the OORs were 2.60 for frequency of binge eating (95% CI = 1.58, 4.41, FDR *p* < 0.001), 6.39 for vomiting (95% CI = 4.06, 10.04, FDR *p* < 0.001), 2.11 for laxatives (95% CI = 1.32, 3.37, FDR *p* = 0.005) and 2.68 for excessive exercise (95% CI = 1.56, 4.46, FDR *p* = 0.001). For comparisons between the untreated and outpatient groups, the OOR for vomiting duration was 1.81 (95% CI = 1.35, 2.43, FDR *p* < 0.001. The group differences on frequency of laxative and excessive exercise were not statistically significant in the model adjusting for lifetime history of AN, suggesting that they were more common in those with a lifetime history of AN, and perhaps accounted for by history of AN.

#### BMI history

The average lowest adult BMI was 23.50 kg/m^2^ (*SD* = 5.77) and the average highest was 35.62 kg/m^2^ (*SD* = 9.92). The inpatient group had a significantly lower illness-related lowest BMI than the outpatient and untreated groups (FDR *p*s < 0.05), but these differences were not significant in the sensitivity analysis after adjusting for lifetime AN. This indicates that those with BN/BED who were treated in inpatient/residential settings were also more likely to have had a history of AN. The outpatient group had a higher lifetime highest adult BMI than the untreated group (FDR *p* = 0.01). In the sensitivity analysis adjusting for history of AN, both inpatient and outpatient groups had higher highest adult BMIs than the untreated group (FDR *p*s < 0.05).

## Discussion

Research studies on eating disorders typically recruit participants from treatment settings, such as hospitals, specialist treatment programs, and healthcare registries, with less recruitment directly from the community. There is a possibility that these samples may not represent those who do not access treatment, which could result in challenges when generalizing medical research findings. This study compared research participants currently symptomatic with BN or BED who self-reported never accessing treatment to those who self-reported accessing treatment at some point in their lives, and found that the former group had a milder illness history and a higher likelihood of being male and identifying as a racial or ethnic minority.

Individuals who reported inpatient or residential care for BN/BED were found to have the most severe illness histories, those who reported outpatient treatment had less severe histories, and those who reported being untreated had the mildest illness history. Clinic-based research may therefore underrepresent individuals with milder illness histories and symptoms. Those who reported treatment reported a younger age at onset of eating disorder behaviors, a history of more prolonged and frequent eating disorder behaviors, greater past-month body dissatisfaction (measured by the EDE-Q Weight Concern), and higher comorbid mental health symptoms. These findings suggest that more severe and longstanding symptoms may motivate help-seeking and correspond with existing research [8–12]. Physical health concerns, such as abdominal pain or other gastrointestinal problems, also prompt help-seeking [34]. DGBIs were very frequently reported (85%) in this sample compared with the general population [35], but were not related to treatment access. Possibly, a ceiling effect may have been operating. Other research has found that patients with eating disorders commonly have gastrointestinal complaints, and receive more referrals to gastroenterology services than other primary care patients [34]. These findings highlight the potential role of gastroenterologists in facilitating access to specialty eating disorder care, although they may require guidance from the eating disorder field to improve their ability to screen and refer people for eating disorders.

We found that vomiting, binge eating, and excessive exercise course, in terms of ever-use, duration, and frequency, distinguished treated and untreated participants. Greater severity of symptoms (e.g., vomiting and binge eating) was associated with self-reported treatment use. Those with higher body dissatisfaction and BMI were also more likely to report using treatment. Some studies have linked these factors to treatment-seeking, whereas others have not [9, 10]. One possible explanation for these discrepancies could be whether eating disorder samples include AN. Some participants were recruited from a digital self-help platform, demonstrating demand for less intensive and more diversified channels of treatment. Digital self-help and eTherapy interventions could be suitable for less symptomatic presentations, and may reduce barriers of cost, geography, privacy concerns, and stigma, and fit within a stepped care model of health service provision [36]. While clinician-led psychotherapy or medication management through doctor’s offices and clinics has been the traditional mode of treatment delivery, the COVID-19 pandemic has led to a dramatic expansion of telehealth services. However, the translation of other types of digital and low-intensity interventions into clinical services and healthcare marketplaces remains limited [37, 38].

There were gender, racial and ethnic disparities in reported treatment access. Males, racial minority individuals, and Hispanic females, were overrepresented in the untreated group. This has been noted in epidemiological research in population-representative samples [13]. Systemic barriers to care, such as economic and insurance issues, often affect racial and ethnic minorities more than others [13]. Gender and race-based stereotypes and stigma also impede access to care. Eating disorders are less readily recognized among males and persons belonging to racial and ethnic minority groups [39, 40]. Fear of and actual unhelpful responses from others, including healthcare practitioners, female-oriented information and services, and internalized masculinity norms (i.e., emotional stoicism, self-sufficiency) may prevent men from disclosing eating disorder symptoms [14, 15]. Males account for 1 in 4 cases of BN and 1 in 3 cases of BED in the general population [41], but they are underrepresented in research and clinical care.

Recruiting individuals from the community into clinical research could improve the representativeness of medical research on eating disorders. Recommendations for recruiting and retaining underrepresented racial and ethnic groups and addressing medical and scientific inequity are rapidly evolving [42]. In some countries, grant funding bodies, federal and state agencies, scientific organizations, and academic institutions are reforming methods from grant review criteria through to hiring and promotional criteria to diversify the scientist workforce. Better health provider and public mental health literacy regarding the occurrence of eating disorders in males, promotion of race- and gender-validated screening tools and outcome measures, culturally sensitive health services that include multilingual therapists, and culturally- and gender-sensitive interventions could promote wider treatment access and representation in clinical research. Further approaches to reduce participation bias in future research include collaborating with community organizations serving underrepresented groups and employing population-based sampling methods.

Limitations of this study include the use of a self-report instrument instead of a clinical interview to capture eating disorder diagnosis. However, computerized algorithms based on DSM-5 criteria generated the AN, BN, and BED lifetime diagnoses, and the ED100K questions were based upon questions used in the SCID, which has been extensively validated. An assessment of current eating disorder diagnosis was not available but would have been ideal, since it includes the psychological components. The questions on ever receiving treatment were designed for the study and may be subject to bias, such as recall bias or if the treatment targeted some other diagnosis besides BN/BED (i.e., AN, purging disorder). While the BEGIN study expanded beyond clinic-based recruitment, it still relied on self-selected participation. Therefore, it remains uncertain whether the sample truly represents the population of those with BN/BED. Treatment access was not verified by health records, and geographical distance from services was unable to be taken into account. Less symptomatic individuals may have faced difficulties in obtaining a diagnosis and insurance coverage for treatment. The sampling approach may have excluded individuals who no longer meet diagnostic criteria after treatment, biasing the treated group toward a more severe presentation. Comparison with BEGIN participants who reported receiving treatment but did not have current BN or BED symptomatology did not support this interpretation. These limitations should be considered when interpreting the results.

In conclusion, this study found that individuals currently symptomatic with BN or BED who reported being untreated, a group that has historically been overlooked in clinical research on eating disorders, reported milder illness histories and were more likely to belong to minority racial and ethnic groups and to be male. More community outreach and recruitment efforts in research are needed to ensure that research evidence translates to all those affected by eating disorders and that our research efforts do not widen medical and healthcare disparities.

## Supplementary Information

**Additional file 1: Table S1.** Comparison of research participants with BN/BED who had never accessed treatment (Untreated) vs those who had accessed treatment (Treated): Sensitivity analysis adjusting for history of anorexia nervosa.** Table S2.** Comparison of research participants with BN/BED by highest level of care received: Sensitivity analysis adjusting for history of anorexia nervosa.

## Data Availability

The Binge Eating Genetics Initiative (BEGIN) dataset has been deposited to the National Institute of Mental Health National Data Archive (NDA) (collection #3361).

## Abbreviations

ADHD: Attention-deficit/hyperactivity disorder
AN: Anorexia nervosa
ASRS-6: Adult ADHD Self-Report Scale v1.1 Screener
BED: Binge-eating disorder
BEGIN: Binge-Eating Genetics Initiative
BMI: Body mass index
BN: Bulimia nervosa
CBT: Cognitive-behavioral therapy
CI: Confidence interval
DSM-5: Diagnostic and Statistical Manual 5^th^ edition
EDE-Q: Eating Disorder Examination Questionnaire
ED100K: Eating Disorders 100,000 Questionnaire
FDR: False discovery rate
GAD: Generalized anxiety disorder
GAD-7: Generalized Anxiety Disorder-7
IBS: Irritable bowel syndrome
IPT: Interpersonal psychotherapy
IRR: Incidence rate ratio
LSMD: Least squares mean difference
MDD: Major depressive disorder
OOR: Ordinal odds ratio
OR: Odds ratio
OSFED: Other specified feeding and eating disorders
PHQ-9: Patient Health Questionnaire-9
RIIIAQ: Rome III diagnostic questionnaire
SCID: Structured Clinical Interview for DSM-5
